# Supplementary figures and images for: FoBSim: an extensible open-source simulation tool for integrated fog-blockchain systems
Source: PeerJ Comput Sci. 2021 Apr 16;7:e431. doi: 10.7717/peerj-cs.431 (PMC8056250; doi:10.7717/peerj-cs.431)

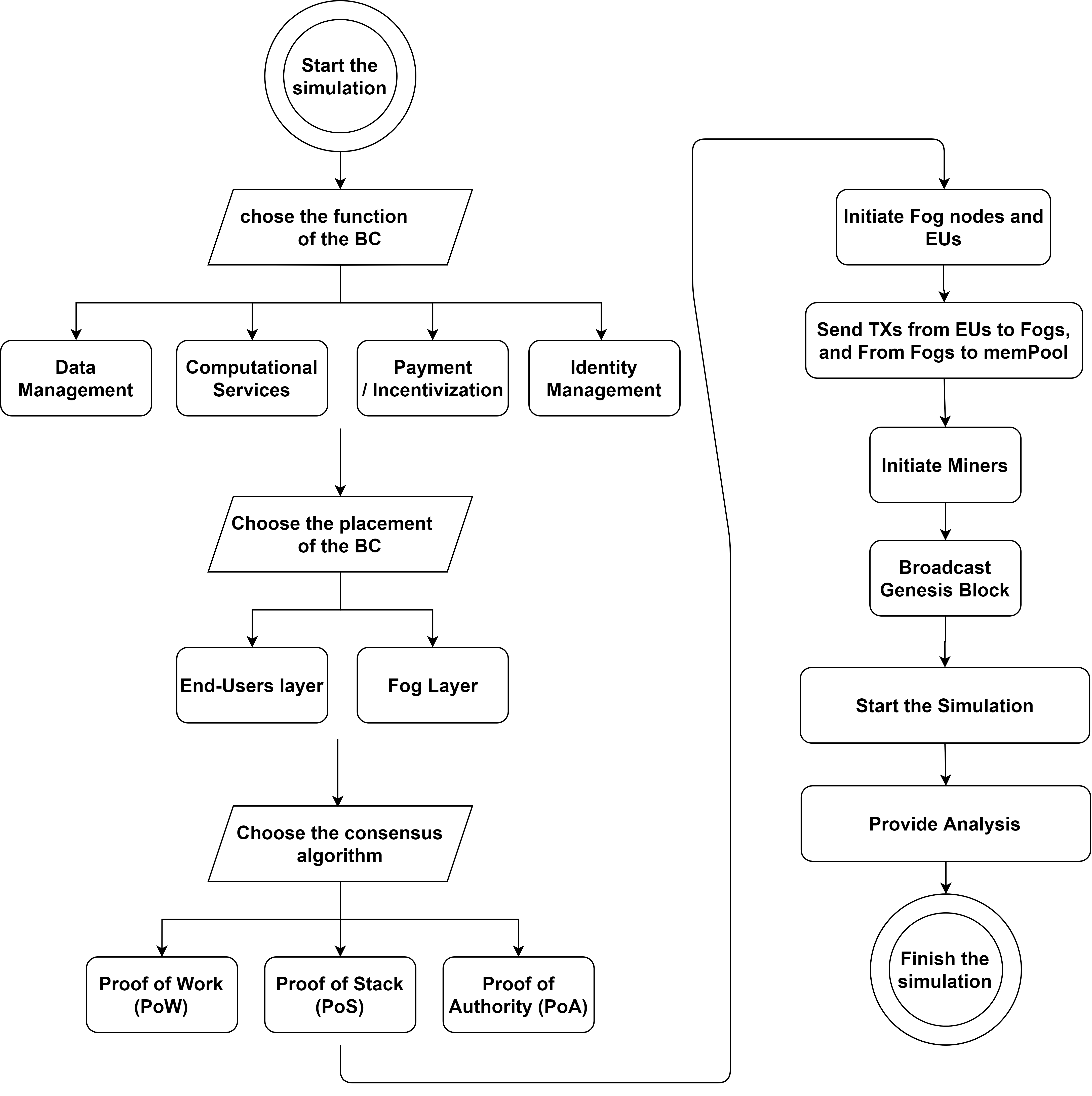

Supplement: Supplemental Information 1 [file peerj-cs-07-431-s001.png]

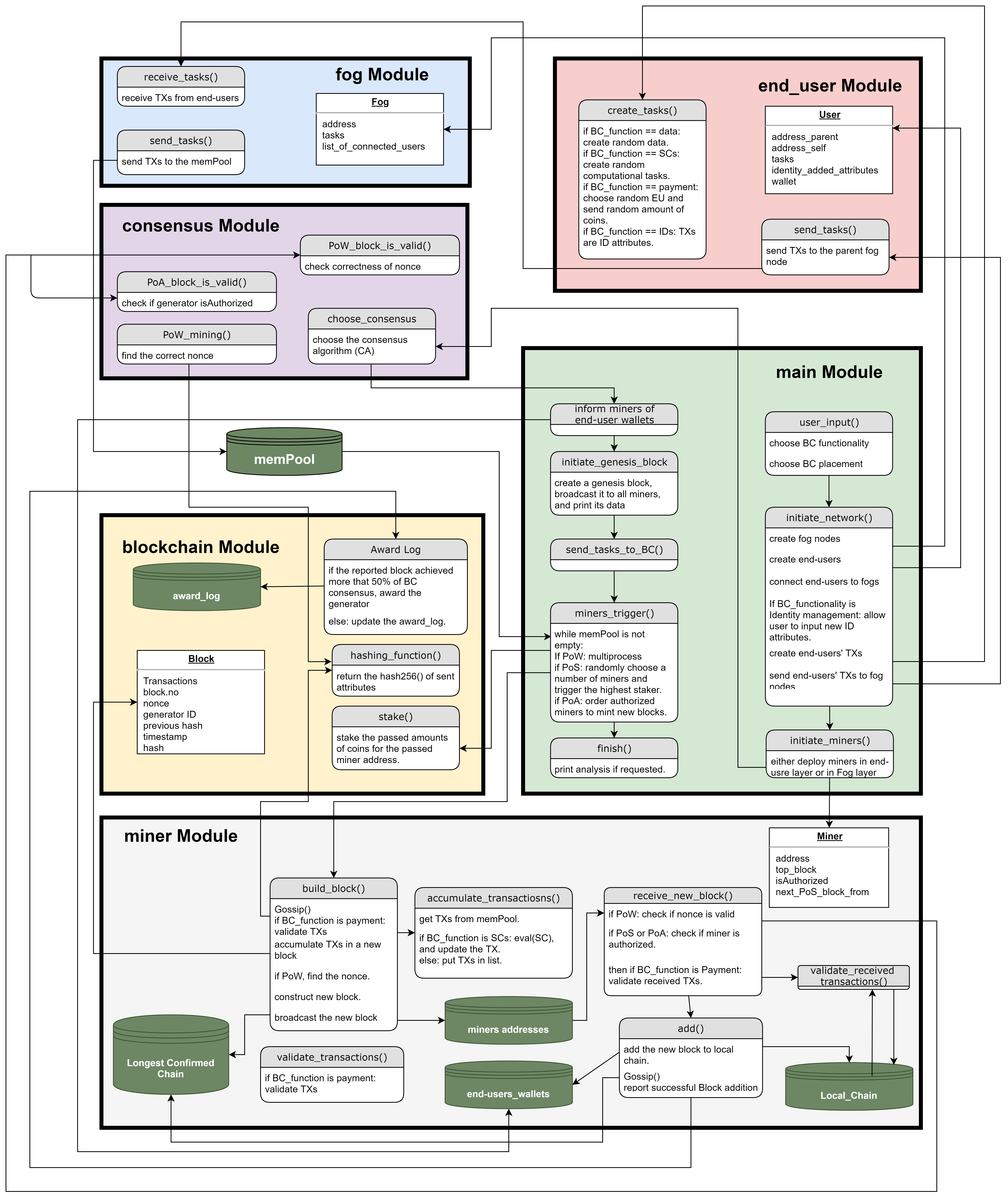

Supplement: Supplemental Information 2 [file peerj-cs-07-431-s002.png]

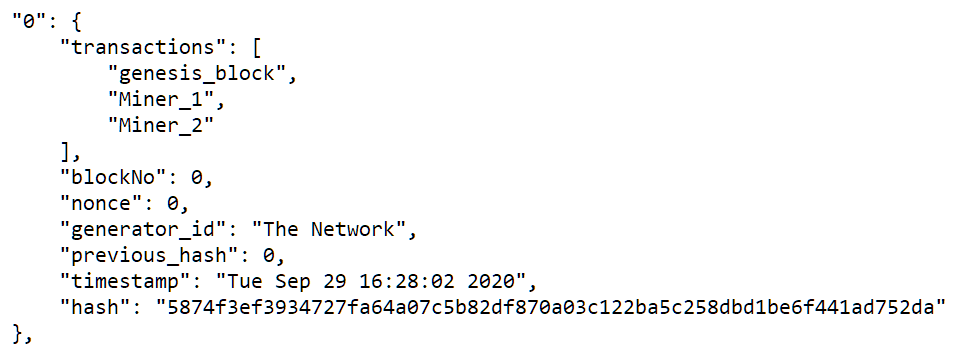

Supplement: Supplemental Information 3 [file peerj-cs-07-431-s003.png]

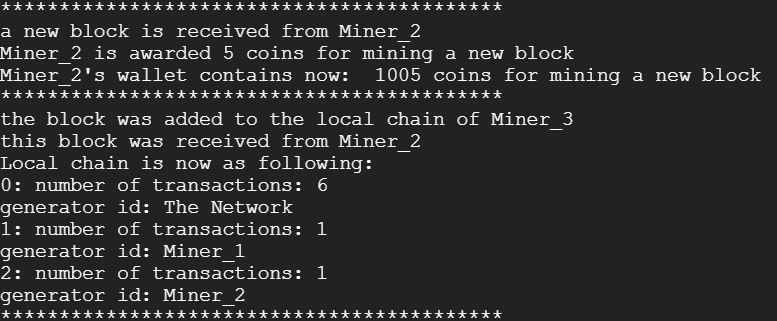

Supplement: Supplemental Information 4 [file peerj-cs-07-431-s004.png]

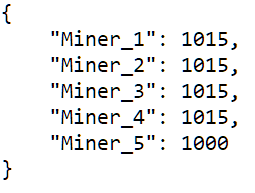

Supplement: Supplemental Information 5 [file peerj-cs-07-431-s005.png]

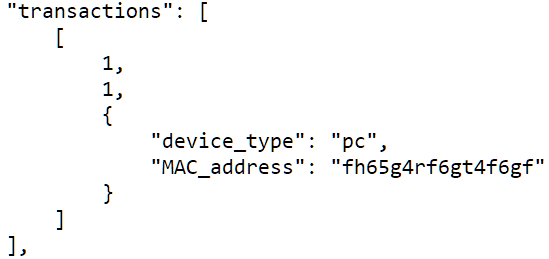

Supplement: Supplemental Information 6 [file peerj-cs-07-431-s006.png]

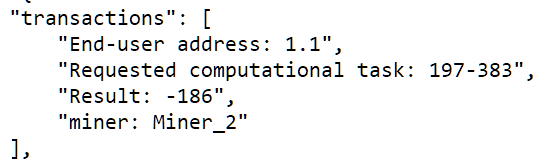

Supplement: Supplemental Information 7 [file peerj-cs-07-431-s007.png]

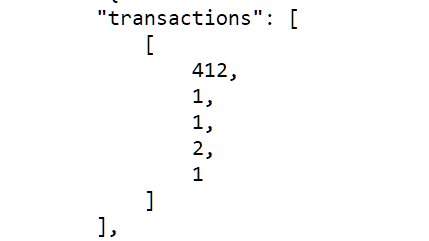

Supplement: Supplemental Information 8 [file peerj-cs-07-431-s008.png]

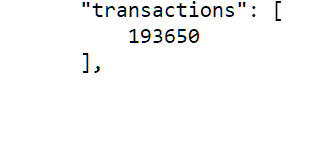

Supplement: Supplemental Information 9 [file peerj-cs-07-431-s009.png]
