# Supplementary material for: FoBSim: an extensible open-source simulation tool for integrated fog-blockchain systems
Source: PeerJ Comput Sci. 2021 Apr 16;7:e431. doi: 10.7717/peerj-cs.431 (PMC8056250; doi:10.7717/peerj-cs.431)
Supplement: Supplemental Information 10 [file peerj-cs-07-431-s010.pdf]

Supplemental Material

FoBSim: An extensible open-source simulation tool for integrated Fog-Blockchain systems

Hamza Baniata and Attila Kertesz

Department of Software Engineering, University of Szeged, Hungary

Corresponding author:
H. Baniata

Email address: baniatah@inf.u-szeged.hu

ABSTRACT

This document provides the Supplemental Material of the paper titled: FoBSim: An extensible open-source simulation tool for integrated Fog-Blockchain systems.

1 APPENDICES

1.1 Figures


```
"0": {
    "transactions": [
        "genesis_block",
        "Miner_1",
        "Miner_2"
    ],
    "blockNo": 0,
    "nonce": 0,
    "generator_id": "The Network",
    "previous_hash": 0,
    "timestamp": "Tue Sep 29 16:28:02 2020",
    "hash": "5874f3ef3934727fa64a07c5b82df870a03c122ba5c258dbd1be6f441ad752da"
},
```


Figure 1. The Genesis block, with all its attributes, generated to miner nodes

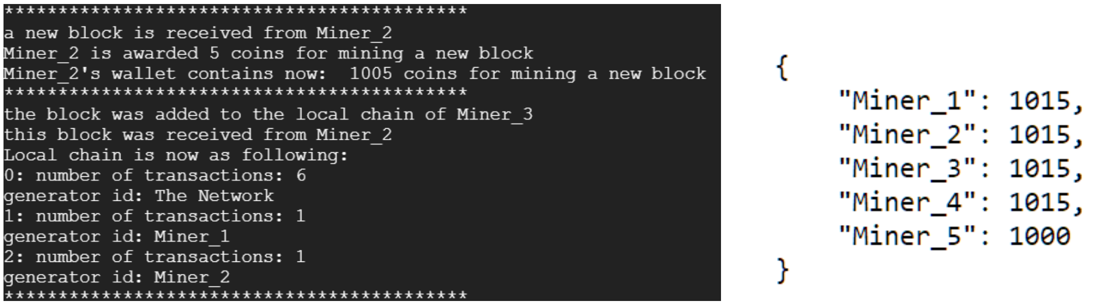

(a) (b)

Figure 2. A sample of FoBSim output. (a) confirming a new block receipt, a new award for mining the new block (as the required percentage of confirmations was reached), and the updated state of local chain of the receiver miner (b) Final miner wallets values in a PoA scenario

```
"transactions": [
    [
        1,
        1,
        {
            "device_type": "pc",
            "MAC_address": "fh65g4rf6gt4f6gf"
        }
    ]
],
```


(a)


```
"transactions": [
    "End-user address: 1.1",
    "Requested computational task: 197-383",
    "Result: -186",
    "miner: Miner_2"
],
```


(b)


```
"transactions": [
    [
        412,
        1,
        1,
        2,
        1
    ]
],
```


(c)


```
"transactions": [
    193650
],
```


(d)

Figure 3. Samples of TXs produced by FoBSim entities (a): BC functionality is Identity Management, (b): BC functionality is Computational Service, (c): BC functionality is Payment, (d): BC functionality is Data Management

1.2 Tables

Function	Description
**user_input()**	The BC_functionality and BC_placement are input by the user. Then this function initiates temporary files. Currently, there are four functionalities available, namely Data management, Computational services, Payment, and Identity management, and two placement options, namely Fog layer and end-user layer.
**initiate_network()**	user inputs additional Id attributes (if applicable). Fogs/end-users are then constructed, end-users are triggered to create new TXs and send them to fogs. Fogs receive TXs and wait for trigger.
**initiate_miners()**	Miners are constructed and relevant temporary files to the BC construction are initiated.
**connect_miners()**	Miners are connected in a P2P fashion and the network is confirmed to be one giant component.
**give_miners _authorization()**	Allows the authorization of some miner nodes to mint new blocks in case the CA is PoA.
**inform_miners _of_users_wallets()**	Informs miners about the initial values of end-user wallets.
**initiate_genesis _block()**	A new block is built whose previous_hash value = 0, block_no = 0, and TXs are the addresses of miners. Then, Fogs are triggered to send TXs in their buffers to mempool.
**miners_trigger()**	Triggers miners to get TXs from memPool and start minting new blocks.

Table 1. Functions in the main.py module

Function	Description
Class: **User**	Initiated with the attributes: addressParent, addressSelf, tasks, identity_added_attributes, and wallet
**create_tasks()**	if the BC function was Data Management, a TX is a randomly generated number coupled with the end-user address. if the BC function was Computational Services, a TX is a randomly chosen Elementary arithmetic operation (i.e. +, -, *, /) coupled with two randomly generated numbers. The produced random computational tasks is coupled with the addresses of end-users. Once a miner solves a computational task, result is appended to the TX, and saved on chain. If BC function is Payment, a TX is a randomly generated amount of coins (up to the amount in the end-user's wallet), coupled with a randomly chosen end-user and the end-user's self address. Validation and confirmation is conducted by the receiver miner. If BC functionality is Identity Management, a TX is the address of the end-user, coupled with any added ID attributes by the user. Table 7 of the appendices declares the four formats of TXs in FoBSim, while Figure 3 of the appendices present screenshots of TXs generated by FoBSim entities.
**add_attributes()**	A function that allows the user to add additional ID attributes to end-user devices.
**send_tasks()**	each user simply sends its tasks to the fog node it is connected with. Note that in FoBSim multiple end-users can connect to one fog node, while each end-user is connected to only one fog node. However, this can be re-configured according to the simulation scenario.

Table 2. The Class and Functions in the end_user.py module

Function	Description
Class: **Fog**	initiated with the attributes: address, tasks, and list_of_connected_users.
**receive_tasks()**	receives the TXs from end-users and saves them in its buffer "self.tasks"
**send_tasks_to_BC()**	sends all TXs in its buffer to the memPool modul

Table 3. The Class and Functions in the Fog.py module

Function	Description
**generate_new _block()**	outputs a list of TXs, a block number, a nonce value, a generator-id, the hash of the previous Block, the timestamp of the generation, and the self hash.
**hashing_function()**	uses the Secure Hash Algorithm (SHA256) to generate the hash of the encoded nonce, TXs, generator-id, and previous hash.
**report_a _successful_block _addition()**	records the votes sent by miners to indicate a successful majority confirmation of a named block.
**fork_analysis()**	A method that, when called, counts the number of different chain versions in the BC network.
**stake()**	used when the PoS algorithm is chosen, where random amounts of coins are taken from each miner's wallet, and staked in the BC. This contributes later to the BC system choosing (randomly) the miner that will mint the next Block, biased by a tendency to choose miners with higher staked coins.
**award_winning _miners()**	reads the voting record of winning miners and adds the winning award to their wallets.

Table 4. Functions in the Blockchain.py module

Function	Description
Class: **Miner**	Initiated with Address, Top_block (for saving the last confirmed block), a Boolean isAuthorized attribute (for declaring whether this miner is authorized to mint new Blocks in a PoA scenario), a next_pos_block_from variable to memorize the address of the next block generator, a set of neighbors, transmission delay, and a boolean gossiping variable.
**build_block()**	constructs valid blocks according to the chosen BC functionality and CA.
**receive_new_block()**	receives new blocks from neighbours, and adds them to its local chain if it was new and valid. When the new block is successfully added, it is forwarded to neighbours, otherwise it is discarded.
**Validate _transactions()**	Accepts new Blocks coming from other miners, validates them according to the BC functionality and the used CA, and adds valid Blocks to the local chain.
**add()**	performs and reports a successful Block addition
**gossip()**	investigates the longest chain in the BC network and, accordingly, updates the local chain according to majority consensus

Table 5. The Class and Functions in the miner.py module

Function	Description
**choose _consensus()**	allows the user to choose one of the available CAs in FoBSim.
**PoW_mining()**	provides miners with the method to search for the puzzle solution in PoW based scenarios.
**PoW_block _is_valid()**	returns either True or False according to the correctness of puzzle solution. If one of the TXs were invalid, the whole Block is rejected.
**PoA_block _is_valid()**	checks the validity of Blocks generated when the PoA CA is chosen. Additionally to the checks performed in the **PoW_block_is_valid()**, this method checks if the miner who minted the block is authorized to do so. If False returned, all TXs within the block are sent back to memPool.

Table 6. Functions in the consensus.py module

**BC functionality**	**TX Format**
Data Management	[random number]
Computational Services	[end-user ID, random computational task, Result, Miner]
Payment	[Amount to be paid, Sender address (parent), Sender address (self), Receiver address (parent), Receiver address (self)]
Identity	[end-user_address(parent), end-user_address(self), Any user added ID attributes]

Table 7. Types and formats of TXs in FoBSim
